# Lessons learned from recruitment and implementation of the Perceived Diet Quality Study

**DOI:** 10.3389/fnut.2026.1938643

**Published:** 2026-10-14

**Authors:** Tameka I. Walls, Alicia S. Landry, Fatemeh Malekian, Gabriela E. Rodriguez, Jessica L. Thomson

**Affiliations:** 1 School of Kinesiology and Nutrition, The University of Southern Mississippi, Hattiesburg, MS, United States; 2 College of Agricultural, Human and Environmental Sciences, Southern University and A&M College, Baton Rouge, LA, United States; 3 Delta Human Nutrition Research Program, USDA Agricultural Research Service, Stoneville, MS, United States

**Keywords:** dietary assessment, enrollment, nutrition research, online surveys, partnering organizations, perceived diet quality, recruitment, reminders

## Abstract

**Background:**

Online surveys offer rapid, cost-effective, and convenient tools for data collection. However, online survey research carries risk, particularly for fraudulent behavior. The objective of this study was to describe lessons learned from implementing an online survey designed to test and validate a comprehensive tool for measuring perceived diet quality.

**Methods:**

Participants were recruited via flyers posted at various community organizations. Recruitment flyers contained a QR code to access the recruitment survey. The study consisted of two surveys, the Perceived Diet Quality Questionnaire (PDQ-Q) and Diet ID^TM^, a dietary assessment, tool. Email reminders were sent to participants who had not completed the surveys within specified time frames. Chi square tests were used to determine whether participant characteristics differed by reminder status (no reminder vs. ≥1 reminder).

**Results:**

Of the 1,337 recruitment surveys submitted, 962 individuals were enrolled in the study, with 605 (63%) and 591 (61%) valid and complete PDQ-Q and Diet ID^TM^ surveys submitted. Majority reasons for not enrolling 375 individuals in the study included duplicate name, email address or telephone number (73%) and suspicious or invalid contact information (15%). The mean number of reminders sent to participants who completed the PDQ-Q and the Diet ID^TM^ was 1.8 (SD = 0.9) and 1.7 (SD = 1.4). For both surveys, the percentage of participants who needed ≥1 reminder increased as age increased (PDQ-Q: 21% for 18–29 years vs. 35% for ≥60 years; Diet ID^TM^: 10% for 18–29 years vs. 22% for ≥60 years).

**Conclusions:**

While QR codes and mobile-first surveys increase accessibility and reduce participant burden, they also introduce risks to data integrity resulting in the need for verification checks. Incorporating verification procedures may reduce redundant submissions and improve efficiency. The observed pattern of diminishing returns after two completion reminders indicates that reminder schedules can be optimized to balance participant engagement. Participant characteristics associated with the need for reminders suggest that fully digital protocols may disadvantage some groups. Future research may involve testing recruitment and retention strategies, evaluating cost-effectiveness of number of email reminders vs. completion percentage, and examining how tailored engagement influences participation among underrepresented populations.

## 1 Introduction

Of the $5.3 trillion spent annually for health care costs in the United States (US), 90% are used to treat individuals with chronic and mental health conditions (1). It is well known that many of these conditions, such as obesity, type 2 diabetes, and heart disease, are tied to poor nutrition; yet the quality of adults' diets in the US remains low (2). Individuals consuming unhealthy foods and beverages must recognize the need to improve their dietary choices before positive behavior changes can be made. However, US adults are not adept at accurately assessing the quality of their diet with just 15% having an accurate perception of their diet (3). Thus, research is needed to determine why there is a disconnect between how healthful individuals believe their diets to be vs. the healthfulness of their actual consumption patterns. To determine if nutrition interventions are successful at closing the gap between perceived and actual diet quality, researchers need a comprehensive and validated tool to measure and track changes in perceived diet quality (PDQ). Thus, the PDQ Study, comprised of online surveys, was designed and implemented to test and validate a comprehensive tool to measure PDQ.

The use of online surveys for health research has grown exponentially in the last decade, particularly during and after the COVID-19 pandemic (4). Online surveys offer fast, cost-effective, and convenient tools for data collection, especially in hard to reach and inadequately depicted populations (5). Further, online surveys have the added benefit of extracting significantly more information on sensitive topics, such as socially undesirable behaviors (6). However, online survey research does not come without risks, particularly for fraudulent behavior. Fraudulent behavior can take many forms including misrepresentation to meet eligibility criteria, repeat responders due to monetary incentives or desire to misinform results for political motivation, and use of sophisticated software applications to automatically fill out surveys (4–7). Hence, implementing strategies to help ensure the scientific integrity of data resulting from online survey research data is necessary. This paper describes the lessons learned from implementing an online survey designed to test and validate a comprehensive tool to measure PDQ.

## 2 Methods

### 2.1 Research design and recruitment

The PDQ Study was a cross-sectional non-experimental survey designed to test and validate the PDQ Questionnaire (PDQ-Q). The PDQ-Q was developed as a comprehensive tool to measure PDQ and determine its associations with dietary intake and other health outcomes. Development of the PDQ-Q involved several steps including focus groups with thematic analysis of the resulting discussions and selection of questionnaire items based on results of the thematic analysis, literature reviews, and exiting surveys. Subsequently, content validity and face validity of the questionnaire were established through expert reviews and cognitive interviews. Finally, internal reliability, criterion reliability, and convergent validity were established for the scale created using factor analysis. Scale items included self-assessed diet quality (qualitative, grade, and numeric), satisfaction with healthfulness of diet, balanced meals, meal planning/preparation, guilt/regret about food choices, and nutrition knowledge. Additional details regarding the development and validation of the PDQ-Q are being reported in another publication (3).

Completion of the PDQ Study occurred in two phases. The first phase consisted of an online survey containing the PDQ-Q, an item about general health, a diet quality questionnaire (8, 9), items about dietary plans/restrictions, reasons for difficulty eating healthy, and a set of demographic questions. The second phase consisted of online dietary data collection via the Diet Quality Photo Navigation (DQPN^®^) technique using the Diet ID^TM^ tool. Briefly, Diet ID^TM^ is a patented image-based algorithm that estimates nutrient intake based on a series of food images and is designed to measure usual dietary patterns over the last 30 days (10, 11). After completion of the recruitment survey, eligible individuals (adults aged ≥18 years) were enrolled into the study and received an email invitation to complete the PDQ-Q using a link embedded in the email. After completion of the PDQ-Q, participants received another email invitation to complete a Diet ID^TM^ assessment using a personalized link embedded in the email. Participants provided electronic informed consent prior to participation in the surveys and received $40 e-gift cards for their time. Data collection occurred from April to September 2025. The PDQ Study was approved by the Institutional Review Board of the Delta State University (protocol 2025–048) and conducted in accordance with the Helsinki Declaration of 1975, as revised in 2008. Individuals were recruited from three southern US states in which the researchers worked—Arkansas, Louisiana, and Mississippi. Recruitment methods included: flyers that were shared with food banks, food pantries and networks, diaper banks (non-profit organization that distributes diapers and other essential hygiene items to families with low income), non-profit community programs, churches, and local health centers, including federally qualified health centers; onsite interactions at recruitment organizations; and word of mouth. Recruitment sites were selected based on the populations they served (i.e., low income) and convenience (i.e., organizations researchers worked with in the past, close proximity to researchers' work sites). Based on *a priori* sample size analysis, a sample size of 425 was deemed sufficient for conducting factor analysis and validating the PDQ-Q (3). Recruitment flyers, in English and Spanish, contained a quick response (QR) code that provided access to the recruitment survey. The electronic recruitment survey and the PDQ-Q were created in English and Spanish using Snap XMP Desktop software version 21.22 (Snap Surveys Ltd., Portsmouth, NH) and data were collected and stored on the Snap WebHost, an online, mobile and secure survey management system. The recruitment survey included six items: confirmation of age (≥18 years), first name, last name, email address, telephone number, and list of recruitment sites to identify how the individual heard about the study. The first four items had to be completed to submit the survey.

Prior to enrolling eligible individuals into the study, a research associate or senior researcher verified that individuals' names, email addresses, and/or telephone numbers were unique. Individuals with the same name, email address, or telephone number as current participants were not enrolled in the study. Additionally, individuals with suspicious email addresses (e.g., same name but different email service provider as current participant) or invalid telephone numbers (e.g., disconnected, no longer in service) also were not enrolled in the study. Eligible individuals' names and email addresses were uploaded into Snap WebHost which was automated to send initial invitations to complete the PDQ-Q followed by two reminder emails sent 1 week apart (i.e., 7 and 14 days after initial invitation). Researchers also had the option to manually send reminder emails. Initial and reminder email invitations for the Diet ID^TM^ assessment were sent manually by a research associate or senior researcher. Additionally, reminder telephone calls were made by a research associate or senior researcher.

Demographic characteristics collected on the PDQ-Q included age (years), sex (male or female), ethnicity (yes or no Hispanic/Latino/Spanish), race, relationship status, education level, work status, and yearly household income. Due to small subsample sizes and analytic purposes, categories were combined as described here. Age included seven categories: 18–19, 20–29, 30–39, 40–49, 50–59, 60–69, and ≥70 years; the first two and last two categories were combined as 18–29 and ≥60 years, respectively. Race included six categories: American Indian or Alaska Native, Asian, Black or African American, Native Hawaiian or Pacific Islander, White, and other. Ethnicity and race were combined as non-Hispanic Black, non-Hispanic White, and other/multiracial. Relationship status included six categories: married, widowed, divorced, separated, never married, and living with partner; categories were combined as married/living with partner, divorced/widowed/separated, and never married. Education level included six categories: ≤ some high school, high school diploma/General Education Development test (GED), some college, associate or technical degree, bachelor's degree, and graduate or professional degree; the first two, middle two, and last two categories were combined as ≤ high school diploma, ≤ 2-year college degree, and ≥bachelor's degree, respectively. Work status included six categories: employed full time (≥30 h/week), employed part time (< 30 h/week), not employed and looking for work, not employed and not looking for work, retired, and disabled and not able to work; the first two and remaining four categories were combined as employed and unemployed, respectively. Income included 12 categories starting with ≤ $25,000 and incrementing by $10,000 until the last category of ≥$125,001; the last eight categories were combined as ≥$55,001. All the demographic items included a “Prefer not to say” response that was coded as missing for analytic purposes.

### 2.2 Analysis

SAS^®^, version 9.4 (SAS Institute, Inc., Cary, NC) was used to conduct analyses. Statistical significance was set at *p* ≤ 0.05. Descriptive statistics were computed for recruitment data (including metadata for survey mode and language), study completion data (including email reminders), and participant characteristics by reminder status for the two study phases. Chi square tests were used to determine whether nominal participant characteristics (sex, ethnicity/race, relationship status, and work status) differed by reminder status (no reminder vs. ≥1 reminder). For characteristics with more than two levels and a significant chi square test, *post hoc* pairwise comparisons were made with Bonferroni corrections applied to the *p*-values (0.05/number of comparisons). Chochran-Armitage trend tests were used to determine whether there was a linear trend in proportions of ordinal participant characteristics (age, education level, and income) by reminder status (no reminder vs. ≥1 reminder). Multivariable logistic regression was used to model the probability of needing ≥1 reminder for PDQ-Q and Diet ID^TM^ assessment, and to compute odds ratios and corresponding 95% Wald confidence intervals for significant effects (explanatory variables). The set of explanatory variables included age, sex, ethnicity/race, relationship status, education level, work status, and income. Explanatory variables were tested for collinearity based on variance inflation factors (>10) and condition indices (>30). Models were fit using complete case analysis; observations with missing data were excluded listwise. Taylor series linearization was used for variance estimation in descriptive analysis where missing values were treated as missing completely at random. Fit and influence diagnostics were performed including assessments of model fit using Hosmer-Lemeshow goodness-of-fit tests and examination of standardized residuals, leverage values, and Cook's distance to identify observations with disproportionate impact on model estimates.

## 3 Results

The study flowchart for recruitment, enrollment, and survey completion is presented in Figure 1. Of the 1,337 recruitment surveys submitted, 375 individuals were not enrolled in the PDQ Study for the following reasons: duplicate name, email address, or telephone number (*n* = 273; 73%), suspicious or invalid contact information (*n* = 57; 15%), no response to verification telephone call (*n* = 33; 9%), study closed (*n* = 10; 3%), declined participation (*n* = 1; < 1%), and ineligibility (*n* = 1; < 1%). Characteristics of the 962 (72%) recruitment surveys that resulted in study enrollment are presented in Table 1. Most surveys were completed via smartphone (96%) and in English (99%). One third (33%) of recruitment occurred through food banks/pantries, followed by health care clinics/centers (10%), and food networks (7%). Of the recruitment organization types at which both passive and active recruitment took place, 84% and 67% of the individuals recruited from food banks/pantries and health care clinics/centers, respectively, were at active sites. The mean time to complete the recruitment survey was 2.3 min [standard deviation (SD) = 4.4].

**Figure 1:**
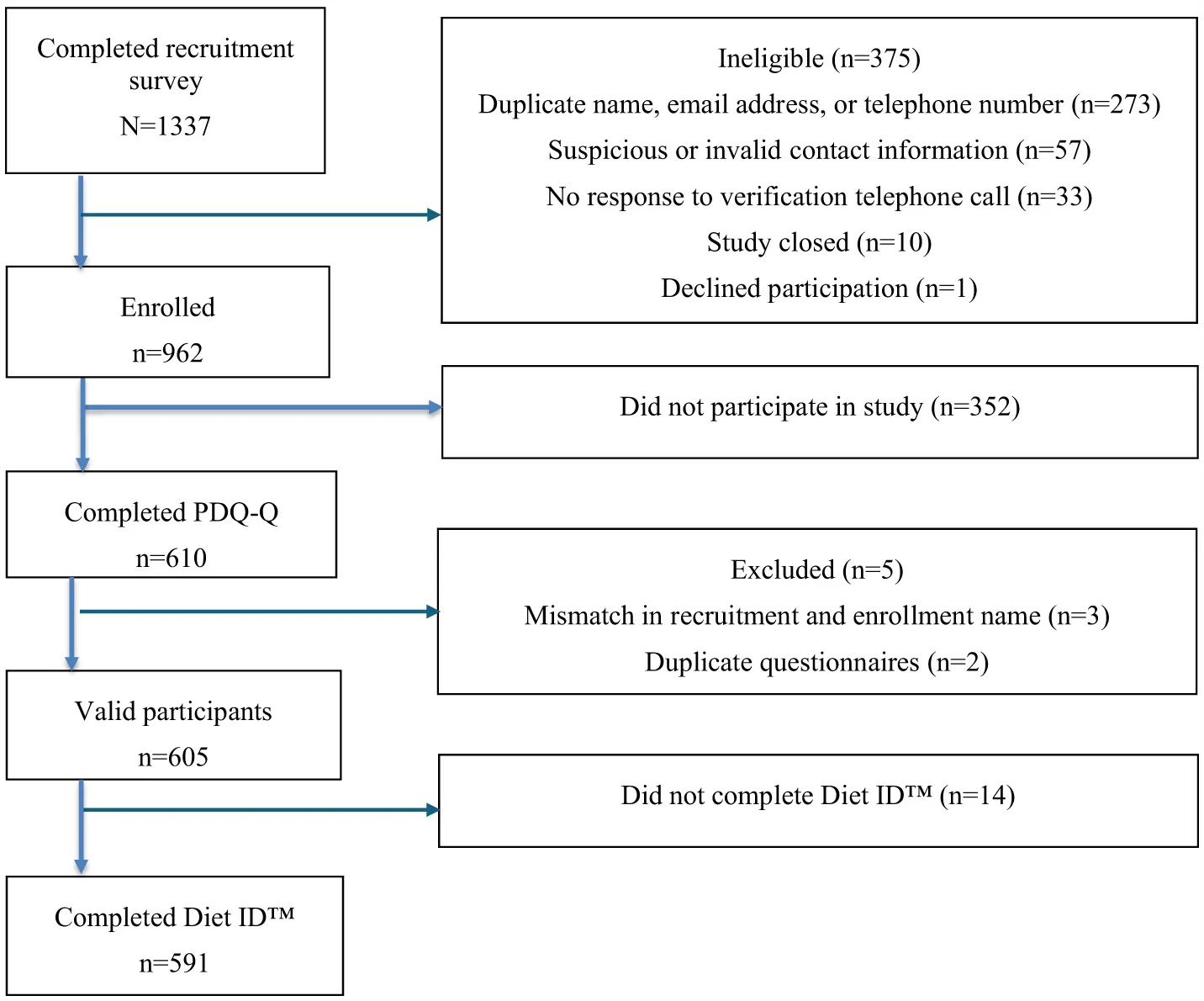
Perceived diet quality study flowchart for recruitment, enrollment, and survey completion.

**Table 1:** PDQ Study recruitment survey results (*N* = 962)^a^.

Item	*n*	%
Survey mode		
Smartphone	922	95.8
Tablet	25	2.6
Personal computer/laptop	15	1.6
Survey language		
English	953	99.1
Spanish	9	0.9
Organization type/mode		
Food bank/pantry^b^	315	32.7
Health care clinic/center3^c^	94	9.8
Food network^d^	64	6.7
Non-profit community program	59	6.1
Word of mouth^e^	56	5.8
YMCA	14	1.5
Flyer	10	1.0
Church	3	0.3
Department of Health	3	0.3
Head Start center	3	0.3
Library	1	0.1
Other-not specified	334	34.7
Missing	6	0.6
	Mean	SD
Time (minutes)	2.3	4.4

SD, standard deviation.

^a^Excludes 375 entries that did not result in study enrollment.

^b^84% (n = 265) of individuals from organizations with prior collaboration or onsite recruitment.

^c^67% (n = 63) of individuals from organizations with prior collaboration or onsite recruitment.

^d^Organization with prior collaboration.

^e^Includes boss, colleague, coworker, family member, friend, individual's name, program specialist, and school.

Of the 962 participants enrolled in the study, 610 (63%) completed the PDQ-Q; however, five questionnaires were excluded due to a mismatch between enrollment and PDQ-Q participant name (*n* = 3) and duplicate questionnaires completed by the same participant using different email addresses (*n* = 2). Of the 605 valid participants who completed the PDQ-Q, 591 (98%) completed the Diet ID^TM^ assessment. For participants who completed the PDQ-Q, the number of reminders sent ranged from 0 to 4 with 76, 11, 8, and 5% of participants completing the PDQ-Q with 0, 1, 2, and ≥3 reminders, respectively. The mean number of reminders sent to participants who completed the PDQ-Q was 1.8 (SD = 0.9). The mean number of days between study enrollment and PDQ-Q completion was 4.5 (SD = 13.9) and the mean time to complete the PDQ-Q was 7.2 min. For participants who completed the Diet ID^TM^ assessment, the number of reminders sent ranged from 0 to 7 with 87, 10, 2, and 2% of participants completing the assessment with 0, 1, 2, and ≥3 reminders, respectively. The mean number of reminders sent to participants who completed the Diet ID^TM^ assessment was 1.7 (SD = 1.4). The mean number of days between PDQ-Q completion and Diet ID^TM^ assessment completion was 2.4 (SD = 5.9) and the mean time to complete the Diet ID^TM^ was 2.5 min.

Comparisons of participant characteristics by PDQ-Q reminder status (no reminder vs. ≥1 reminder) are presented in Table 2. The percentage of participants who needed ≥1 reminder significantly increased as age increased (21% for 18–29 years vs. 35% for ≥60 years). Additionally, participants who needed ≥1 reminder were significantly more likely to be female than male (27 vs. 17%). Further, the percentage of participants who needed ≥1 reminder significantly decreased as income increased (31% for ≤ $25,000 vs. 17% for ≥$55,001).

**Table 2:** Participant characteristics by PDQ-Q reminder status (*N* = 605).

Characteristic	No reminder		≥1 reminder		*p* ^a^
	*n*	%	*n*	%	
Age, years (*N* = 601)					0.0016
18–29	147	79.5	38	20.5	
30–39	131	82.9	27	17.1	
40–49	79	72.5	30	27.5	
50–59	52	69.3	23	30.7	
≥60	48	64.9	26	35.1	
Sex (*N* = 597)					0.0083
Male	143	83.1	29	16.9	
Female	310	72.9	115	27.1	
Ethnicity/race (*N* = 501)					0.1168
Non-Hispanic Black	258	78.4	71	21.6	
Non-Hispanic White	83	69.2	37	30.8	
Other/multiracial	38	73.1	14	26.9	
Relationship status (*N* = 559)					0.3210
Married/living with partner	182	72.8	68	27.2	
Divorced/widowed/separated	88	80.0	22	20.0	
Never married	152	76.4	47	23.6	
Education level (*N* = 586)					0.7989
≤ High school diploma	142	79.8	36	20.2	
≤ 2-year college degree^b^	146	69.5	64	30.5	
≥Bachelor's degree	155	78.3	43	21.7	
Work status (*N* = 579)					0.1348
Employed (full or part-time)	315	77.2	93	22.8	
Unemployed^c^	122	71.3	49	28.7	
Income (*N* = 516)					0.0273
≤ $25,000	92	69.2	41	30.8	
$25,001–$35,000	81	81.8	18	18.2	
$35,001–$45,000	48	68.6	22	31.4	
$45,001–$55,000	40	74.1	14	25.9	
≥$55,001	133	83.1	27	16.9	

PDQ-Q, Perceived Diet Quality Questionnaire.

^a^P-value for chi square test or Cochran-Armitage trend test (age, education level, and income).

^b^Includes some college but no degree and Associate or technical degree.

^c^Includes not employed and looking for work, not employed and not looking for work, retired, and disabled and not able to work.

Comparisons of participant characteristics by Diet ID^TM^ assessment reminder status (no reminder vs. ≥1 reminder) are presented in Table 3. The percentage of participants who needed ≥1 reminder significantly increased as age increased (10% for 18–29 years vs. 22% for ≥60 years). Additionally, participants who needed ≥1 reminder were significantly more likely to be married/living with partner and divorced/widowed/separated than never married (17% and 17 vs. 8%), and significantly more likely to be unemployed than employed (19 vs. 12%).

**Table 3:** Participant characteristics by Diet ID^TM^ reminder status (*N* = 591).

Characteristic	No reminder		≥1 reminder		*p^a^*
	*n*	%	*n*	%	
Age, years (*N* = 588)					0.0030
18–29	162	90.5	17	9.5	
30–39	137	88.4	18	11.6	
40–49	96	88.1	13	11.9	
50–59	58	80.6	14	19.4	
≥60	57	78.1	16	21.9	
Sex (*N* = 584)					0.9393
Male	147	87.0	22	13.0	
Female	360	86.7	55	13.3	
Ethnicity/race (*N* = 493)					0.2556
Non-Hispanic Black	291	89.5	34	10.5	
Non-Hispanic White	98	83.8	19	16.2	
Other/multiracial	45	88.2	6	11.8	
Relationship status (*N* = 549)^b^					0.0118
Married/living with partner	205	83.3	41	16.7	
Divorced/widowed/separated	89	83.2	18	16.8	
Never married	181	92.3	15	7.7	
Education level (*N* = 573)					0.8497
≤ High school diploma	149	87.6	21	12.4	
≤ 2-year college degree^c^	175	85.4	30	14.6	
≥Bachelor's degree	172	86.9	26	13.1	
Work status (*N* = 567)					0.0158
Employed (full or part-time)	354	88.5	46	11.5	
Unemployed^d^	135	80.8	32	19.2	
Income (*N* = 505)					0.6277
≤ $25,000	110	87.3	16	12.7	
$25,001–$35,000	85	87.6	12	12.4	
$35,001–$45,000	56	80.0	14	20.0	
$45,001–$55,000	48	90.6	5	9.4	
≥$55,001	141	88.7	18	11.3	

^a^P-value for chi square test or Cochran-Armitage trend test (age, education level, and income).

^b^Pairwise comparisons indicated never married significantly different from married/living with partner (p = 0.0047) and from divorced/widowed/separated (p = 0.0143) at Bonferroni corrected significance level of 0.0167.

^c^Includes some college but no degree and Associate or technical degree.

^d^Includes not employed and looking for work, not employed and not looking for work, retired, and disabled and not able to work.

No evidence of multicollinearity was present in the set of explanatory variables used for logistic regression (variance inflation factors < 1.4 and condition indices < 23). Hosmer-Lemeshow test was non-significant (*p* > 0.4) suggesting model fit was adequate and influence diagnostics did not show evidence of observations with disproportionate impact on model estimates. Results of the multivariable regression models are presented in Table 4. For the PDQ-Q, age, relationship status, and income were significant explanatory variables. Compared to participants aged 30–39 years, participants aged 40–49, 50–59, and ≥60 years had 2.3 (0.44^−1^), 2.7, and 3.6 times the odds, respectively, of needing ≥1 reminder. Participants who were married/living with partner and never married had 2.5 and 2.2 times the odds, respectively, of needing ≥1 reminder than participants who were widowed/divorced/separated. Participants with household incomes ≤ $25,000 and $35,001-$45,000 had 2.1 and 2.8 times the odds, respectively, of needing ≥1 reminder than participants with household incomes ≥$55,001; whereas participants with household incomes $35,001-$45,000 had 2.6 times the odds compared to participants with household incomes $25,001-$35,000. No explanatory variables were significant in the Diet ID^TM^ model.

**Table 4:** Multivariable logistic regression results for needing ≥1 reminder to complete surveys.

Model and variable^a, b^	OR	95% CI		*P* ^c^	LRT^d^	Gamma^e^	c-statistic^f^
PDQ Questionnaire					0.0009	0.40	0.70
Age (years)				0.0351			
18–29 vs. 30–39	1.53	0.76	3.09				
18–29 vs. 40–49	0.68	0.33	1.40				
18–29 vs. 50–59	0.56	0.25	1.28				
18–29 vs. ≥60	0.43	0.19	1.01				
30–39 vs. 40–49	0.44	0.21	0.93				
30–39 vs. 50–59	0.37	0.16	0.85				
30–39 vs. ≥60	0.28	0.12	0.68				
40–49 vs. 50–59	0.82	0.36	1.87				
40–49 vs. ≥60	0.64	0.27	1.49				
50–59 vs. ≥60	0.78	0.32	1.91				
Relationship status				0.0323			
Married/LWP vs. DWS	2.54	1.25	5.15				
Married/LWP vs. NM	1.13	0.63	2.03				
NM vs. DWS	2.22	1.05	4.76				
Income				0.0321			
≤ $25k vs. >$25k–$35k	1.98	0.90	4.36				
≤ $25k vs. >$35k–$45k	0.76	0.35	1.65				
≤ $25k vs. >$45k–$55k	0.96	0.42	2.24				
≤ $25k vs. >$55k	2.14	1.02	4.49				
>$25k–$35k vs. >$35k–$45k	0.39	0.16	0.91				
>$25k–$35k vs. >$45k–$55k	0.49	0.19	1.24				
>$25k–$35k vs. >$55k	1.08	0.48	2.42				
>$35k–$45k vs. >$45k–$55k	1.26	0.52	3.10				
>$35k–$45k vs. >$55k	2.81	1.32	5.98				
>$45k–$55k vs. >$55k	2.22	0.95	5.19				

PDQ, perceived diet quality; OR, odds ratio; CI, confidence interval; LWP, living with partner; DWS, divorced/widowed/separated; NM, never married; k, 1,000.

^a^Covariates included age, sex, race/ethnicity, education, marital status, work status, and household income.

^b^Model not significant for Diet ID^TM^ assessment.

^c^P-value for multivariable logistic regression model effect.

^d^P for chi-square likelihood ratio test.

^e^Liberal asymmetric measure of ordinal association.

^f^Measure of model's predictive power (equivalent to area under the receiver operating characteristic curve).

## 4 Discussion

This study describes lessons learned from implementing online surveys designed to test and validate a comprehensive tool to measure PDQ. Table 5 presents a summary of challenges encountered, lessons learned, and implications for future studies. While we believe that the PDQ Study was well designed and planned, not all aspects of the study's implementation went as smoothly as anticipated. In the areas of the study for which we had firm control (enrollment and data collection), only minor issues occurred (e.g., duplicate questionnaire submission). In the area of the study for which we had less control (recruitment), we were challenged more than expected. Organizations with the largest proportion of recruits were either those we worked with previously (food banks, food networks, and health care clinics/centers) or at which we conducted onsite recruitment (food banks and health care clinics/centers). Organizations with the lowest proportion of recruits were those at which flyers were permitted for display, but no formative contact was made with the organization's personnel (e.g., YMCA, churches, libraries). Thus, prior contact or collaboration with referral sources and onsite recruitment were essential to our recruitment efforts. In a study involving cancer patients and their caregivers, onsite recruitment yielded the highest number of recruits, while flyers posted at places frequented by cancer patients (e.g., hospitals) yielded the lowest number of recruits (12). Similarly, a study highlighting the continuum of community engagement model found that trust building efforts facilitated participant engagement (13). Therefore, sustained community partnerships may promote trust and enhance participation, particularly when established prior to conducting research in populations of interest. However, in a study involving American Indian tobacco users, advertisement and onsite recruitment at community events resulted in approximately equal numbers of recruits (84 vs. 82) although more recruits from onsite sources enrolled in the study (14). Of note in the current study, an unexpected source of recruitment was “word of mouth” referrals by friends, family members, and coworkers. It is possible that this referral method, known as snowball sampling, allowed us to reach a larger proportion of individuals with low income, although the risk of selection bias cannot be ruled out as referrals are likely to come from similar social circles and therefore certain subgroups may be missed (15).

**Table 5:** Lessons learned from implementation of the PDQ study.

Area	Challenge encountered	Lesson learned	Implications for future studies
Recruitment	Lower than expected enrollment at some organizations	On site recruitment improved recruitment rates	Engage community partners early and involve in study design
Recruitment	Duplicate entries	Instructions to submit survey only once needed on flyers	Provide more information on recruitment flyers including expected time frame for study enrollment
Data integrity	Duplicate entries, invalid contact information	Calling individuals helped to rule out duplicate entries and potential infection by internet robots (bots)	Use proven strategies for detecting bots and fraudulent responses (e.g., CAPTCHA, attention checks, consistency checks, IP address verification)
Staff workload	Manually sending email reminders required significant amount of time	Sending more than 1–2 reminders did not substantially increase completion rate	Use automated system for email reminders when possible; if not possible, send only one reminder
Reminder system	Older, lower income, unemployed and female participants relied more heavily on reminders	Multi-channel reminders may be essential to support diverse populations	Offer reminders via email, text, or telephone calls; offer telephone survey/interview for questionnaires

CAPTCHA, completely automated public turing test to tell computers and humans apart; IP, internet protocol.

Another recruitment challenge involved the use of a QR code on the recruitment flyer. While the QR code made providing contact information convenient and easy for recruits, it resulted in a substantial number of duplicate entries in the recruitment data. The duplicate entries created unexpected, time-consuming work to identify and verify whether individuals were already enrolled in the study. During telephone call verification, the most common reason given for submitting multiple surveys was to ensure that their survey was received. Although we typically enrolled eligible individuals within 1–3 days of receiving their contact information, adding an expected time for study enrollment (e.g., within 5 working days) to recruitment flyers may help prevent some duplicate entries during recruitment. Additionally, requiring a valid telephone number for submission of the recruitment survey was essential for ruling out duplicate entries (e.g., same phone number but different email address) as well as helping to safeguard against internet robots (bots). Recruits who did not provide valid telephone numbers (e.g., disconnected, no longer in service) were not enrolled in the study. Although calling recruits was time-consuming, we believe the effort was essential for helping ensure the integrity of the study data. While not used in the current study, additional safeguards against bots include using Completely Automated Public Turing test to tell Computers and Humans Apart (CAPTCHA), attention checks (special questions added to a survey to see if a participant is reading the directions), consistency checks (comparison of two or more related questions to see if the answers make sense together), and IP address verification. However, these additional safeguards may unintentionally disadvantage certain groups (e.g., individuals with low literacy or cognitive impairment). Hence, care needs to be taken to balance data integrity with inclusivity.

For enrollment and data collection, very few issues occurred (e.g., duplicate questionnaire submission). We believe this was largely due to the use of an online mobile and secure survey management system that automated participant enrollment, email invitations and reminders for completing the PDQ-Q, and data collection. The completion rate for the PDQ-Q (63%) was similar to rates reported in other online surveys (51%−70%) (16, 17). In comparison, the completion rate for the Diet ID^TM^ assessment was substantively higher (98%). We believe the high rate for the Diet ID^TM^ assessment was mostly due to participants receiving an invitation for the assessment after completion of the PDQ-Q (interested participants) and receiving their monetary incentive after completion of the Diet ID^TM^ assessment (motivated participants). Alternatively, the higher completion rate for the Diet ID^TM^ assessment compared to the PDQ-Q may have been due to the shorter time required to complete the surveys (approximately 3 vs. 7 min). However, this explanation is difficult to substantiate given the Diet ID^TM^ assessment was completed after the PDQ-Q and hence there is no comparative group that received the Diet ID^TM^ assessment first.

For reminders, two reminders appeared optimal for the PDQ-Q while one reminder was optimal for the Diet ID^TM^ assessment in terms of diminishing completion rates (i.e., ≥95% completed the surveys with one and two reminders). However, because reminders were automated for the PDQ-Q, sending more than two reminders (e.g., four) did not result in extra work and therefore might be worth the small increase in completion rate. Whereas reminders were sent manually for the Diet ID^TM^ assessment and thus sending more than the one reminder may not be worth the small increase in completion rate. Based on the mean time between enrollment and PDQ-Q completion (4.5 days), sending reminders every 4–5 days (vs. 7 days) may have helped increase its completion rate. Likewise, sending a reminder for the Diet ID^TM^ assessment 2–3 days after the initial invitation may have helped increase its completion rate.

Older age, being married/living with partner or single, and lower income in general were characteristics associated with survey completion with reminders based on multivariable logistic regression analysis. While these findings are interesting, they are somewhat counterintuitive. We expected that older age and having lower income would have resulted in faster completion rates (i.e., before reminders were sent) because of greater financial motivation. No studies in the literature could be found to support these findings, although an online workplace lifestyle intervention did not find any demographic differences between participants who completed a baseline questionnaire with and without reminders (18). It is possible that the positive associations between needing study reminders and older age and having lower income are at least partly due to retirement. A study comparing older (aged ≥65 years) employed adults to older retired adults found that being retired was associated with more negative attitudes toward technology and less online self-efficacy (19). Thus, it may be that individuals who are retired need more encouragement to participate in and complete online survey studies compared to younger employed individuals with higher income. Offering alternative methods for sending reminders, such as via text or telephone calls, may help reduce the number of reminders needed for completion. Additionally, offering questionnaire completion by telephone interview may help improve completion rates among participants who have limited digital access or literacy or are less comfortable with technology. The associations between needing reminders and relationship status are intriguing although we could find no studies in the literature to support or refute these findings. In the study comparing older employed adults to older retired adults, being married was associated with more negative attitudes toward technology and less online self-efficacy (19). Clearly, more research is needed to determine if these associations are valid and why the differences may exist.

Some limitations of the study bear mentioning. The study data was self-reported and thus may be subject to bias. Participants represented convenience samples of individuals residing in three states; hence results may not be generalizable to these three states nor the general US population. However, the inclusion of participants from more than one state was a strength of the study. Characteristics of individuals who were enrolled in the study but did not participate in the study were not captured. These individuals may represent specific demographic groups that are resistant to participating in online research even though they expressed interest by completing the recruitment survey. Because of potential contamination by internet robots (bots), online research should be considered exploratory, although the current study included safeguards to minimize and identify bot infection (e.g., checking for valid email addresses and/or valid telephone numbers). However, these safeguards may have unintentionally disadvantaged certain populations such as those with lower income or limited resources leading to frequent changes in telephone numbers, use of prepaid cell phones, or sharing of telephones/cell phones. Alternative verification methods that may reduce exclusion while maintaining data integrity include email verification using one-time codes and physical mail verification using postcards with unique codes.

## 5 Conclusions

Findings from this study offer several practical lessons learned from recruitment and methodological implications for conducting web-based nutrition research, particularly among populations that include individuals with lower socioeconomic status. Our results contribute to implementation science literature as they underscore the value of relationship-centered recruitment strategies where partnerships with trusted community organizations seemed more effective than passive approaches (e.g., flyers alone). This suggests that future studies targeting similar populations should prioritize sustained collaborations and in-person presence. Embedding recruitment within service delivery settings also may enhance equity by improving access for individuals who may be less likely to engage in research through traditional online advertising or institutional channels. Through community-based participatory research, which emphasizes partnering with trusted service delivery settings to promote equitable access and reduce structural barriers, individuals may be more willing and able to engage in health research.

The number of duplicate or invalid recruitment submissions (*n* = 375, 28%) highlights important considerations for digital enrollment. While QR codes and mobile-first surveys increase accessibility and reduce participant burden, they also introduce risks to data integrity and potentially increased staff workload to identify duplicate respondents. Incorporating verification procedures, such as required phone numbers and automated duplication checks, may reduce redundant submissions and improve efficiency. The observed pattern of diminishing returns after two reminders suggests that reminder schedules can be strategically optimized to balance participant engagement. Participant characteristics associated with the need for reminders also carry important implications. Older age and lower income were linked to greater reliance on reminders, indicating that fully digital protocols may disadvantage some groups. These findings point to the need for design strategies that proactively address these barriers such as capturing preferred contact mode at enrollment, offering live support (call-back line), and offering questionnaire completion by telephone survey/interview. By examining barriers and facilitators to online research recruitment and data integrity, results of this study add to implementation science by helping to close the gap between what is known to work and what gets done in practice. Future research may involve formally testing recruitment and retention strategies and evaluating cost-effectiveness of different strategies as well as examining cost-effectiveness of multiple email reminders (for non-automated systems) vs. completion percentage. Further, research examining how tailored engagement approaches influence participation among underrepresented populations, particularly those with chronic health conditions, cognitive impairment, and low digital literacy, is needed.

## Data Availability

The data supporting the findings of this study are openly available in USDA Ag Data Commons at 10.15482/USDA.ADC/32207928.v1.
